# The Sensation Seeking Scale (SSS-V) and Its Use in Latin American Adolescents: Alcohol Consumption Pattern as an External Criterion for Its Validation

**DOI:** 10.5964/ejop.v13i4.1198

**Published:** 2017-11-30

**Authors:** Vanina Schmidt, María Fernanda Molina, María Julia Raimundi

**Affiliations:** a National Council of Scientific and Technique Research (CONICET), Buenos Aires, Argentina; b Research Institute, Faculty of Psychology, University of Buenos Aires, Buenos Aires, Argentina; Webster University Geneva, Geneva, Switzerland

**Keywords:** sensation seeking, adolescence, novelty seeking, risk

## Abstract

Sensation Seeking is a trait defined by the seeking of varied, novel, complex, and intense situations and experiences, and the willingness to take physical, social, and financial risks for the sake of such experience. The Sensation Seeking Scale (SSS-V) is the most widely used measure to assess this construct. In previous studies a variety of psychometric limitations were found when using the SSS-V with Latin American population. The purpose of this study is to present additional psychometric properties for its use with Latin American adolescents. It was applied to a 506 adolescent sample (from 12 to 20 years). The result is a scale of 22 items that cover four factors. It seems that sensation seeking among Latin American adolescents can be described in terms of four factors, but with some slightly content differences from what is usually found in adult samples from other countries. Future lines of research are proposed.

Sensation Seeking (SS) is “a trait defined by the seeking of varied, novel, complex, and intense sensations and experiences, and the willingness to take physical, social, legal, and financial risks for the sake of such experience” (Zuckerman, 1994, p. 27). Previous studies have identified biological and behavioural correlates of SS showing that this variable is a strong determinant of attitudes, interests, behaviours and habits. Early in 1969, Zuckerman proposed that SS was based on an optimal level of stimulation in terms of arousal. Years later, a psychobiological model was presented changing the concept of the optimal level of arousal to an optimal level of catecholaminergic activity (Zuckerman, 2007a). Thus, a group of monoamines, enzymes and metabolites interacting in a complex way might be the biological substrate of SS (Zuckerman, 1984, 1989, 2007a).

From Darwinist perspective, SS could be seen as a “stable evolutionary strategy” (Dawkins, 1976), a behaviour that evolved through millions of years of evolutionary history, because it represents an adaptive advantage.

The SS construct has been related to various psychological traits such as impulsivity, dominance, surgency, autonomy, extraversion (as defined by Eysenck, 1967), psychoticism (as re-defined by the same author in his last formulation; Eysenck & Eysenck, 1994), disinhibition (Pelechano-Barberá, 2000; Zuckerman, 1994; Zuckerman, Eysenck, & Eysenck, 1978), and it is negatively related to conscientiousness (Costa & McCrae, 1992) showing in sensation seekers a tendency towards impulsivity and nonconformity (Zuckerman, 2007b). It has been related as well to cognitive and perceptual styles (Glicksohn, Naftuliev, & Golan-Smooha, 2007), attention tasks (Brocke, Beauducel, & Tasche, 1999; Zuckerman, 1990), and different types of experiences with sex (Donohew et al., 2000; McCoul & Haslam, 2001), risk sexual behaviour (Newcomb, Clerkin, & Mustanski, 2011) extreme sports (Michel et al., 1999; Zuckerman, 1984), gambling (McDaniel & Zuckerman, 2003; Morris & Griffiths, 2013; Powell, Hardoon, Derevensky, & Gupta, 1999), driving (e.g. Peer & Rosenbloom, 2013; Smorti, 2014), risk perception (Hampson, Severson, Burns, Slovic, & Fisher, 2001; Horvath & Zuckerman, 1993), risk behaviour (Hansen & Breivik, 2001), and to high risk professions like firemen, police and lifeguards (Wismeijer & Gomà-i-Freixanet, 2012; Zuckerman, 1994).

Numerous researchers and theorists have noted that SS (or related constructs) are the basis for many types of antisocial behaviour and a variety of behavioural disorders such as alcoholism (Zuckerman, 1990), criminality (Zuckerman, 1989, 1994), pathological gambling (Zuckerman, 1994, 2005), agressive behaviour (Zuckerman, 1989), antisocial personality disorder (Gabel, Stadler, Bjorn, Shindledecker, & Bowden, 1994; Zuckerman, 1989).

A topic of widespread concern in our region is the consumption of alcohol, being the risk factor that contributes most to the total burden of noncommunicable diseases in Latin American population (World Health Organization [WHO], 2000). International research has concluded that high SS subjects have a greater potential for being alcohol users and abusers because they constantly seek new experiences (González-Iglesias, Gómez-Fraguela, Gras, & Planes, 2014; Latorre Román, Cámara Pérez, & García Pinillos, 2014; Roberti, 2004; Zuckerman, 1990). It is probably that in Latin America samples SS trait is among the strongest correlates of alcohol use and misuse.

All of the above shows the relevance of this construct and, therefore, of having the appropriate instrument for its assessment.

In a wide range of researches differences between sex and ages were found. In England (Zuckerman & Neeb, 1980), USA (Zuckerman, 1994), Australia (Roberti, 2004), Canada (Ridgeway & Russell, 1980), Spain (González-Iglesias et al., 2014; Pérez & Torrubia, 1986), France (Michel et al., 1999), Germany (Brocke et al., 1999), men get higher scores in SS total score. To summarize, males get higher scores in SS and most of its dimensions than females, pointing out a pancultural feature (Cross, Cyrenne, & Brown, 2013; Smorti, 2014).

Associations with age are interesting because they describe a curvilinear relationship: SS increases with age from adolescence to adulthood and then decreases as the person gets older (Zuckerman & Neeb, 1980). In a Spanish sample with adolescents it was found that age was positive related to different dimensions (subscales) of SS and SS total score (Latorre Román et al., 2014). No Latin American study about differences in SS according to sex and age is available.

Given this background, it is important to study sex and age differences as additional contributions to construct validity.

The *Sensation Seeking Scale Form V* (SSS-V; Zuckerman et al., 1978 revised by Zuckerman, 1994) is the most widely used self-report standardized measure to assess this construct. Factor analyses (Zuckerman, 1971) yielded four factors that allowed the developing of four subscales (extracted from Zuckerman, 1979):

- Thrill and Adventure Seeking (TAS) consists of items expressing desires to engage in sports or activities involving some physical danger or risk such as mountain climbing, parachute jumping, scuba diving, speeding in a car, etc.
- Experience Seeking (ES) contains items describing the desire to seek new experiences through mind and senses by living in a nonconforming lifestyle with unconventional friends, and through travel.
- Disinhibition (DIS) was the name used for the items describing the need to disinhibit behaviour in the social sphere by drinking, partying and seeking variety in sexual partners.
- Boredom Susceptibility (BS) items indicate an aversion for repetitive experiences of any kind, routine work, or even dull or predictable people. Other items indicate a restless reaction when things are unchanging.

Zuckerman (1994) proposed that TAS is the non impulsive-socialized way of SS and ES, DIS and BS are impulsive-unsocialized ways of SS.

Form V of the SSS was a shortened version of Form IV (Zuckerman, 1971) and was developed by using large heterogeneous samples from England and North America. On the basis of the cross-sex and cross-national similarities in item loadings, the new Form V was constructed. This form contains 10 items representing each of the four factors and uses a Sensation Seeking Total Score based on the sum of the four factor scores (Zuckerman, 1979). In the 1980´s the author became aware of the datedness of some of the items and changed the wordings or explained some terms in the item itself (Zuckerman, 2007a). Thus, a revised version appeared in his second book on SS (Zuckerman, 1994).

The SSS-V was translated into many languages and adapted to many countries and it is used in a variety of cultures. New versions were also devised to study SS in children and adolescents in different cultures. A junior SSS (J-SSS) was adapted in Spain (Pérez, Ortet, Plá, & Simó, 1986) using the SSS form V (Zuckerman et al., 1978), translated into French and used for subjects aged 11-15 years (Simó, Santana, & Pérez, 1991). No factor analysis was performed for these versions. SSS for French adolescents (14-19 years) was devised (Michel et al., 1999) using the French abbreviated form of SSS (Carton, Jouvent, & Widlöcher, 1992). The SSS for children (SSSC) was based on 72 items from the adult version of the SSS (Zuckerman & Neeb, 1980) and was devised for children aged 7-12 years (Russo et al., 1991). The second version of SSSC included additional items and was completed by children aged 9-15 years (Russo et al., 1993). A Swedish scale inspired in Zuckerman´s scales was devised for adolescents around age 14 (Hansen & Breivik, 2001). Another Spanish adaptation was presented by Pérez and Torrubia (Pérez & Torrubia, 1986) but no factor analysis was performed. For Italian population, a recent study with a large sample analyzed the internal structure of the SSS- V by EFA and CFA, finding a two-factor structure (TAS and DIS) (Manna, Faraci, & Como, 2013). What is clear from these adaptations is that there are slightly differences from culture to culture in the way SS expressed itself among adolescents (see Table 1).

**Table 1:** Comparative Table of Different Versions of SSS-V for Adolescents

Country / adaptation data	Non impulsive SS	Impulsive SS		
England, USA Zuckerman et al. (1978; Zuckerman, 1994) 18 yrs. or older	TAS: Risk sports and other physic activities (risky but socially accepted)	DIS: Desinhibition through alcohol consumption, parties and a variety of sexual experiences	ES: Seek for novelty experiences through nonconventional life style (including drug consumption but not exclusively)	BS: aversion to the familiar, repetitive activities, people and predictable routine
France Russo et al. (1993) 9-15 yrs.	TAS: Risk sports exclusively	Social DIS: Desinhibited behaviour in social (specially, peers context)	AAD: Positive attitude towards alcohol and illegal drugs	
France Michel et al. (1999) 14-19 yrs.	TAS: Risk sports exclusively	Social DIS	ES (very similar to original ES)	NC: Nonconformism, nonconventional life style (similar to original ES but including original BS)
Sweden Björck-Åkesson (1990) 12-16 yrs.	TAS: Risk sports exclusively	ACT (Activity). Measures the urgency to perform tasks quickly; impulsivity in social context (similar to DIS)	NES: Novelty seeking, impulsivity and lack of control (similar to ES)	OUT: Measures the desire to be the center of attention (contains ES and BS items)
Italy Manna et al. (2013) 13-24 yrs.	TAS: Risk sports or other physically risky activities involving speed or danger.	DIS: seeking sensation through social activities, such as parties, social drinking and variety in sexual partners		

For Latin American population, measures of SS are not adequately developed and validated (they have been merely translated). Furthermore, SS in adolescents may differ from SS in adults, as some studies have suggested (Michel et al., 1999; Russo et al., 1993; Schmidt, 2008). A SSS for Latin adolescents is, therefore, needed and has to be adapted and validated. Present research was designed to fill this void by presenting additional data on validating process of SSS-V for its use in Latin American adolescent population.

In previous studies (Messoulam, Abal, & Molina, 2005; Schmidt, Messoulam, & Molina, 2008; Schmidt, Messoulam, Molina, & Abal, 2006; Schmidt, Molina, Messoulam, & Abal, 2004) although good psychometric properties were found, content and metric analyses showed problems with some items. More than 85% of adolescents answered negatively to Item *32. It is better if two married persons begin their sexual life together* and to Item *22. I stay away from anyone I suspect being “gay” or “lesbian”...* showing low discriminant capacity in both cases. Moreover, adolescents expressed their surprise and annoyance with both items. We think it was due to both items were outdated for our context and so they do not represent sensation seeking trends. Finally, we considered that in countries where wide sectors of the population have difficulties in getting daily nutritional sustention it was not convenient nor ethical to ask if they “*like to try new foods*” as in Item 14. That is why Items 14, 22 and 32 were removed. Other items needed to be revised (Items 12, 16, and 19) because for our adolescents some words had no sense (“punk”, “swingers” and “water-skiing”). Even though some of the items from the original version (1978) had been slightly modified by the author using terms more relevant to current times (Zuckerman, 1994), we found in our previous studies that some difficulties still persisted. Even after translating them, our adolescents could not imagine such activities. Similar problems were found in other adaptations, specifically with BS factor (Chico-Librán & Vázquez-Orellana, 1999; Manna et al., 2013; Ridgeway & Russell, 1980). We proposed then modifications to these three items (for example, in Item 16, “water skiing” was changed to “car race pilot” because they could have a clearer idea of what it was), and the words “swinger” and “punks” were omitted (instead of swingers, in Item 12 we talked about people who are uninhibited and free about sex and instead of punks, in Item 19 we talked about making friends in not conventional groups). Finally, we generated three new ones as we had lost three items and two additional ones in case some of the three items did not work properly. In Table 2 we present the first pull of items developed to create the Latin American version of SSS and we compare them with the original ones.

**Table 2:** Linguistic and Conceptual Adaptation (Items Modified, Removed and New Items)

*Original Items*		*Items for Latin America Version*
1.	I like “wild” uninhibited parties	Original item
2.	I can´t stand watching a movie…	Original item
3.	I often wish I could be a mountain climber	Original item
4.	I like some of the earthy body smells	Original item
5.	I get bored seeing the same old faces	Original item
6.	I like to explore a strange city…	Original item
7.	When you can predict almost everything…	Original item
8.	I usually don´t enjoy a movie or play where I can predict…	Original item
9.	I have tried marijuana…	Original item
10.	I would like to try some of the drugs that produce…	Original item
11.	I sometimes like to do things…	Original item
***12.***	***I enjoy the company of real “swingers”***	*I would enjoy the company of very liberal people with respect to sex.*
13.	I often like to get high…	Original item
14.	I like to try new foods…	ITEM REMOVED
15.	Looking at someone´s home movies, videos…	Original ítem
***16.***	***I would like to take up the sport of water skiing***	*I would like to be a car racer.*
17.	I would like to try surfboard riding	Original item
18.	I would like to take off on a trip with no preplanned…	Original item
***19.***	***I would like to make friends (…) like artists or “punks”***	*I would like to have friends in some strange groups.*
20.	I would like to learn to fly an airplane	Original item
21.	I would like to go scuba diving	Original item
22.	I would like to meet some persons who are homosexual (men or women)	ITEM REMOVED
23.	I would like to try parachute jumping	Original item
24.	I prefer friends who are excitingly unpredictable	Original item
25.	I like to have new and exciting experiences…	Original item
26.	I often find beauty in the “clashing” colors…	Original item
27.	I get very restless if I have to stay around…	Original item
28.	I like to dive off the high board	Original item
29.	I like to date person who are physically…	Original item
30.	Keeping the drinks full is the key…	Original item
31.	The worst social sin is to be a bore	Original item
32.	A person should have considerable sexual experience…	ITEM REMOVED
33.	I could conceive of myself seeking pleasures…	Original item
34.	I like people who are sharp and witty even if…	Original item
35.	I enjoy watching many of the “sexy” scenes in movies	Original item
36.	I feel better after taking a couple of drinks	Original item
37.	People should dress in individual ways…	Original item
38.	I would like to sail a long distance in a small…	Original item
39.	I have no patience with dull or boring persons…	Original item
40.	I think I would enjoy the sensations of skiing…	Original item
41.		*I often find attractive bright and strong colors. (new item)*
42.		*I would try drugs that have strange or dangerous effects. (new item)*
43.		*I like to take risks for fun. (new item)*
44.		*I like some strong odors. (new item)*
45.		*I like the noise and the bustle around me. (new item)*

*Note.* Boldface indicates modifications in original items and italic indicates new items created for Latin America version.

In the present work, we decided to expand the sample and study the indicators of validity and reliability of the scale in greater depth, using currently recommended analysis and methods (Lloret-Segura, Ferreres-Traver, Hernández-Baeza, & Tomás-Marco, 2014).

## Method

### Participants

It was a convenience sample. Adolescents (*n* = 506) with a *mean* age of 14.96 (*SD* = 1.62; rank = 12-20) from four schools from Buenos Aires Metropolitan Area (Buenos Aires city and Buenos Aires Province) completed the survey voluntarily and anonymously. Informed consent forms were provided and completed by parents and students. The sample consisted of 314 females (62.10%). They lived in biparental homes (49.50%), monoparental homes (22.27%), extended families (12.52%), and the rest of them in some of the other forms of family composition.

### Instruments

- *Socio-demographic Questionnaire* to explore sex, age, grade at school, and family composition.
- *Sensation Seeking Scale Form V* (*SSS-V*; Zuckerman et al., 1978; revised by Zuckerman, 1994; Latin American adaptation: Schmidt, 2008; Schmidt et al., 2004). In previous studies developed in Argentina (Schmidt, 2008; Schmidt et al., 2004, 2006) a detailed content analysis including back-translation procedure, expert opinions, adolescent opinions, and a study of item metric properties when applying the scale to large samples were performed. Some items were modified, others removed and new items had to be developed (see Table 2). The adapted version consisted of 42 items with a forced choice form (subjects have to choose between two possibilities or different situations, one representing a high willingness to SS and the other, the opposite tendency). It assesses the four aspects of SS: Thrill and Adventure Seeking (TAS), Experience Seeking (ES), Disinhibition (DIS), and Boredom Susceptibility (BS).
- *Frequency x Quantity Alcohol Consumption and Heavy Episodic Drinking (HED) Questionnaire.* It was devised following the quantity-frequency approach and based on previous studies (Cremonte, Cherpitel, Borges, Peltzer, & Santángelo, 2010). In this questionnaire the amount (e.g. When you take an alcoholic drink, how many drinks do you usually jacks on each occasion?), frequency (e.g. During the last twelve months/last month how often did you take at least one drink?), and heavy episodic drinking (HED) is assessed following graduated frequency approach (e.g., how often did you take six or more drinks on one occasion?). To distinguish low-risk drinkers from high-risk drinkers we consider both parameters: frequency and quantity. High-risk adolescents drinkers consume alcohol “1 or 2 times a week” or more (category called “frequent consumption”) and/or six or more Drink Units (DU = 60 grams of pure ethanol or more) (category called “heavy episodic drinkers”). This questionnaire has proved to be adequate in our context. Strong relationships between high-risk drinkers and unsocialized ways of SS are expected to be found, providing additional construct validity to the SSS argentine version for adolescents.

### Data Analyses

An Exploratory Factor Analysis (EFA) through Classic Parallel Analysis (PA) was applied (Horn, 1965). Unweighted Least Squares (ULS) for determining the number of factors to retain was applied (Lorenzo-Seva, 1999). This method works on a policoric matrix and is strongly recommended in an asymmetric distribution and with dicotomic response option scales (Muthén & Kaplan, 1992). Promin (Lorenzo-Seva, 1999), a method for oblique factor rotation, was used as correlation among factors is expected. Matrix adequacy was assessed by KMO test and Bartlett esphericity test. The GFI was used to assess the adequacy of a four factor model. The criterion of loading chosen for retaining each item into each factor was greater than .30 (Hair, Anderson, Tatham, & Black, 2001). For double loading, a combined criterion that considers relevant to maintain conceptual consistency and psychometric quality was preferred (Lloret-Segura et al., 2014). Percentage of variance for each factor and the total scale were calculated. Alphas coefficients for each factor and for the total scale were also calculated. Intratest policoric correlation is presented.

Non parametric test were performed as the distribution of the scores was not normal. Males and females were compared on each subscale and the total score using Mann-Whitney *U* Test. The relationship between age and SS was explored with Spearman correlation. Disciminant validity with regard to different alcohol consumption patterns was analyzed with Mann-Whitney *U* Test. Intratest differences (Wilcoxon Test) were performed. We study size effect with Spearman’s *rho.* Statistical analysis was conducted using the Factor program (9.3.1 version) (Lorenzo-Seva & Ferrando, 2015) and the SSPS program (21 version).

## Results

EFA results showed several items with low or double loadings. It was therefore necessary several iterations procedures, removing at each step the items with worse functioning, until both pursued conditions emerged: theoretical consistency and a metrically solid structure (Lloret-Segura et al., 2014). Four factors were extracted that together explained the 56.18% of the variance. The KMO and Barrett test showed the adequacy of the matrix (Table 3). In every factor there are adequate loadings (items with loadings lower than .30 were eliminated). Two items have double loading. Following Lloret-Segura et al. (2014) criteria, we considered double loading when the difference between items is below .20. Statistical and conceptual criterion was used to decide which item goes to which factor. For one item, a conceptual criterion was preferred without resignation of metric quality and, in the other case the criterion followed was statistical (retaining the item in the factor with the highest loading).

**Table 3:** Exploratory Factor Analysis of SSS-V for Adolescents

Item	Factor^a^			
	I	II	III	IV
10. Surfing	**.74**	-.07	-.02	-.14
12. Flying Plane	**.79**	-.04	.09	-.12
13. Diving	**.54**	-.01	.01	.03
14. Skydiving	**.66**	.05	-.04	.30
16. Jumping from Heights	**.81**	.19	-.18	-.10
21. Skiing	**.41**	-.23	.15	.14
1. Wild Parties	-.09	**.40**	.14	.31
8. Disinhibited People	.05	**.37**	.39	-.08
17. Exciting People	.04	**.69**	-.17	-.01
19. Sexual Content Movies	.25	**.36**	.12	-.13
20. Alcohol	-.04	**.84**	.23	-.29
2. Body Smells	-.05	-.12	**.42**	.03
5. Marihuana	.04	-.11	**.99**	-.07
6. Hallucinogens Drugs	.01	.06	**.90**	-.08
9. Stimulants	-.08	.27	**.60**	.17
22. Dangerous Drugs	-.10	.34	**.46**	.14
3. Unpredictable Places	.11	-.04	.02	**.48**
7. Risk Appetite	.04	-.11	.13	**.79**
11. Unplanned Trip	.14	.01	.03	**.41**
15. Unpredictable Friends	.06	.21	-.09	**.36**
18. Boredom Rejection	-.24	.28	-.10	**.48**
23. Risk for Fun	.02	.27	.05	**.44**
Eigenvalues	6.58	2.89	1.58	1.31
% of Variance	29.91	13.13	7.19	5.96
Reliability estimate	.87	.82	.93	.77

^a^*KMO* = .83, Bartlett’s test: *χ*^2^(231, *N* = 506) = 2075.90 (*p* < .001). Total explained variance = 56.18%.

*Note.* Boldface indicates the highest factor loadings for each factor.

The result is a scale of 22 items that cover four factors. It seems that sensation seeking among Latin American adolescents (from 12 to 20) can be described in terms of four factors, similar to those found in adult version from other countries, but with some important differences in the content of each factor. Two factors are similar to TAS and DIS original factors (there are slight differences as it will be shown below). ES is now a dimension clearly assessing illegal drugs attitudes. And a new factor has emerged, which we have named Risk and Novelty Seeking.

### Factor´s Description

Factor I “Thrill and Adventure Seeking” (6 items): this factor reflects the desire to engage in sports or activities involving some physical danger or risk such as surf boarding, flying an airplane or scuba diving. All items are from the original TAS subscale. This dimension was shown to be relevant for adolescents in other adaptations (Michel et al., 1999; Russo et al., 1993). Although some items may vary, in every SSS there is a factor referred to outdoor sports and other experiences involving danger but all being socially acceptable activities and this is the factor with better psychometric properties through different adaptations.

Factor II “Disinhibition” (5 items): this factor assesses the tendency towards disinhibited behavior through parties, sex, alcohol, seeking sexually exciting partners. Disinhibited behaviour (social and sexual) seems to be an important way of SS for our adolescents.

Factor III “Experience Seeking” (5 items): this factor reflects the willingness to seek new and varied experiences through senses, specially through psychoactive substances and through smells. It keeps 5 items from the original ES subscale. The tendency to seek novelty and intense sensations is expressed in this factor through the use (or intention to use) illegal drugs such as marijuana, hallucinogens, stimulants; but it is also expressed through the fondness of feeling strong smells. Thus, a basic sensorial aspect seems to be closely related to the use of illegal drugs.

Factor IV “Risk and Novelty Seeking” (RNS, 6 items): this factor contains items from the original ES subscale, but also items from BS, TAS and new items were developed because of the problems detected in previous studies. Although they belong to different factors (looking back to Zuckerman’s scale), there is a content homogeneity. The items show the preference for a risky lifestyle full of novelty and variety, exploring strange places and taking off on no preplanned trips, and being involved in risky or frightening activities just for fun. We consider this factor might be assessing a general willingness to experience sensations through novelty and risky situations, so as to avoid boredom susceptibility. That is why we re-named this factor.

As it usually happens in other standardizations (Australia, Canada, USA, Spain, Italy) TAS is the subscale which receives the highest score (Zuckerman, 1994, 2007b). The second more preferred subscale is NRS. And ES and DIS received the lowest scores. Descriptive statistics for subscales are presented in Table 4.

**Table 4:** Descriptive Statistics for Each Subscale

Subscale	*M*	*SD*	CI 95%	Min.	Max.
TAS	4.19	1.70	[4.03, 4.33 ]	0	6
ES	1.25	1.48	[1.11, 1.37]	0	5
DIS	1.67	1.37	[1.55, 1.79]	0	5
RNS	2.87	1.68	[2.72, 3.02]	0	6
Total SS	9.98	4.37	[9.59, 10.36]	0	21

*Note.* TAS = Thrill and Adventure Seeking; ES = Experience Seeking; DIS = Disinhibition; RNS = Risk and Novelty Seeking; TSS = Total Sensation Seeking.

The associations analyses among subscales shows that TAS is positively associated to all the other ways of SS, but with low to medium coefficients (*r*_S_ from .14 to .34). The highest association is with RNS (*r*_S_ = .34; *p* < .001). ES is positively associated to all other ways of SS, with moderate coefficients with DIS (*r*_S_ = .49; *p* < .001) and RNS (*r*_S_ = .39; *p* < .001). RNS is positively associated to all the other ways of SS with moderate coefficients (*r*_S_ from .34 to .41) (Table 5).

**Table 5:** Spearman Correlations Among Subscales and Correlations With Age

Variable	1	2	3	4	5	6
1. TAS	-	.14**	.18***	.34***	.62***	.04
2. ES		-	.49***	.39***	.67***	.22***
3. DIS			-	.41***	.70***	.08
4. RNS				-	.78***	.04
5. TSS					-	.11**
6. Age						-

*Note.* TAS = Thrill and Adventure Seeking; ES = Experience Seeking; DIS = Disinhibition; RNS = Risk and Novelty Seeking; TSS = Total Sensation Seeking.

*p < .05. **p < .01. ***p < .001.

Correlation with age shows that SS total score is positively associated to age. Among subscales, the only one positively associated to age was ES. Males got higher scores than females in TAS (*U* = 23917, *p* < .001, *r* = .18), DIS (*U* = 21623, *p* < .001, *r* = .24), RNS (*U* = 25706.50, *p* = .005, *r* = .12), and SS Total Scale (*U* = 22139, *p* < .001, *r* = .22). There is no significant difference in ES.

**Figure 1:**
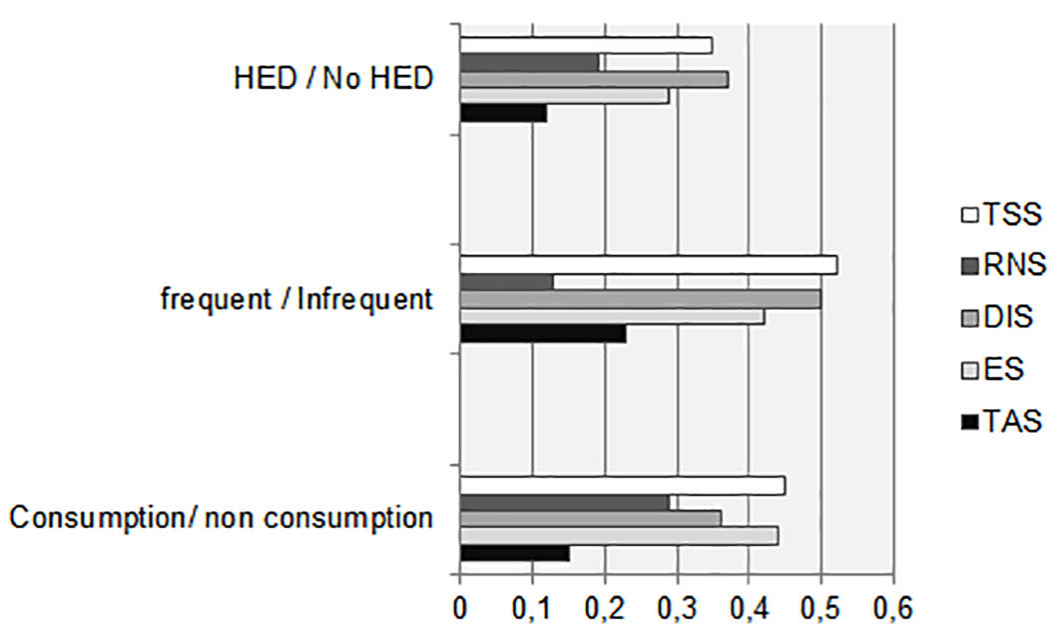
Effect sizes of the difference in sensation seeking, according to the pattern of alcohol consumption. *Note.* HED = Heavy episodic drinking; RNS = Risk and novelty seeking; DIS = Disinhibition; ES = Experience seeking; TAS = Thrill and adventure seeking.

The group of adolescents that consume alcohol showed greater TAS (*U* = 5534.50, *p* = .025, *r* = .15), ES (*U* = 3328.50, *p* < .001, *r* = .44), DIS (*U* = 3885.50, *p* < .001, *r* = .36), RNS (*U* = 4391, *p* < .001, *r* = .29) and overall SS (*U* = 3131, *p* < .001, *r* = .45) than adolescents that had never consumed this substance. Adolescents who at the time of the survey consumed alcohol once or more a week got higher scores in TAS (*U* = 1557, *p* = .009, *r* = .23), ES (*U* = 1055, *p* < .001, *r* = .42), DIS (*U* = 3885.50, *p* < .001, *r* = .50) and SS Total Score (*U* = 818, *p* < .001, *r* = .52) than adolescents who consumed alcohol once a month or even less. No difference in RNS was found (*U* = 1778.50, *p* = .129, *r* = .13). Heavy episodic drinkers (6 or more DU in the same occasion) showed greater scores in ES (*U* = 1374.50, *p* = .001, *r* = .29), DIS (*U* = 1181, *p* < .001, *r* = .37), RNS (*U* = 1651, *p* = .025, *r* = .19) and general SS (*U* = 1215.50, *p* < .001, *r* = .35) than adolescents who used this substance moderately (less than 6 DU). No difference in TAS was found (*U* = 1846.50, *p* = .160, *r* = .112). ES, DIS and Total SS showed the biggest effect sizes (see Figure 1).

## Conclusion and Discussion

Sensation Seeking Scale (*SSS-V*; Zuckerman et al., 1978; revised by Zuckerman, 1994) is the most widely used measure to assess SS, but there was not a complete and adequate adapted version for its use in Hispanic cultural context. Therefore, a new version of Zuckerman´s SSS was required to assess SS in Latin American adolescents. This local version shows adequate reliability, content and construct validity, and expected sex and age differences.

The EFA identified a four-factor structure similar to that found in many adolescent and adult scale versions (England, USA, Spain, Canada, Australia) (Zuckerman, 1994) but with some differences with regard to factor´s contents. TAS and DIS remain almost the same for Latin American adolescents compared to adolescent and adult versions from others countries (Michel et al., 1999; Russo et al., 1993). But ES in our version is different from ES in adult versions. It is clear that for our adolescents the way of seeking experiences and intense sensations is mainly through illegal, dangerous and/or unknown drugs. In Russo et al. (Russo et al., 1993) version, similarly, a third factor emerged: Drug and Alcohol Attitudes. This third factor is similar to our ES factor although in our version the alcohol item was placed in DIS subscale (as in the original version).

Boredom Susceptibility (the fourth factor in the original version) was the most problematic factor, as the loading values for the items were low or did not coincide in the same factor; furthermore, a clear factor similar to BS never emerged in the different solutions that were tried. This factor happens to be the most problematic one in other adaptations too (Manna et al., 2013; Michel et al., 1999). For example, in the Italian adaptation, 14 items failed to meet the retention criteria and were eliminated and a two-factor structure was found (TAS with 7 items from the original TAS subscale and DIS with 5 items from the original DIS subscale). In our version a fourth factor emerged, not comparable to any factors from adolescent or adult versions. The focus for this fourth factor is on risky and new situations and original items related to homosexual friends, artist friends, original clothes, etc. did not show loadings in this factor. Therefore, this factor represents seek of novelty and risky experiences through a variety of situations like travelling, having exciting friends, being involved in frightening activities, and avoiding predictable experiences or friends. We called this facet “Risk and Novelty Seeking” (RNS).

To sum up, sensation seeking in our culture may be well represented through four aspects (as it occurs in other countries) but these aspects are not just the same as those found in other contexts. The scale we finally obtained is different from SSS-V used in England and USA, and different from previous adaptations conducted in Spain (Pérez et al., 1986), France (Michel et al., 1999; Simó et al., 1991) and Italy (Manna et al., 2013).

Relations between subscales (coefficients from low to moderate) suggest that we are assessing different aspects of the same construct, so it is important to consider this facets´ differentiation when assessing this phenomenon. ES and DIS are two clearly impulsive ways of SS, with higher association coefficients between them than with the rest of the subscales. TAS, a non-impulsive way of SS, is mostly associated to BRN, which is probably another non-impulsive way of sensations seeking in our context.

In the present study, males get higher scores in SS total score and in almost every subscale than females. Similar results regard differences according to sex were obtained in England: differences in TAS, DIS, BS and SS Total Scale were found in students at University of Delaware (data from 1986-1992; *n* = 410 males, *n* = 807 females) (Zuckerman, 1994); and in Spain: men obtained greater scores again in TAS, DIS, BS and SS Total Scale. No differences were observed for the ES scores (Chico-Librán & Vázquez-Orellana, 1999). In USA samples, differences in TAS and DIS were also found (males higher than females). In Australia, Canada and Spain, men got higher scores again in SS total score, TAS and BS than women (Roberti, 2004). In Germany, males score higher than females for the SSS-V total score, ES, DIS and BS (Brocke et al., 1999). In SSS for French adolescents, males got greater scores in TAS than females and girls got greater scores in Non-conformism (a factor obtained in their adaptation) (Michel et al., 1999). The lack of differences in ES for males and females may be showing a cultural phenomenon. In the last years, the prevalence of women consuming different substances is rising in the Americas, reaching men levels of consumption for almost every substance (WHO, 2015).

In the present study it was found that age was positively related to ES through illegal drugs. This result is also observed in epidemiological studies (WHO, 2015) and in cross cultural research all around the world (Arnett, 2012), and may be showing an adolescent normal growing process through which the individual gets more chances to do outdoors activities. The decreasing parental control and concomitant increasing independence and autonomy facilitate unconventional, risky and even illegal experiences (Arnett, 2012).

Differences found in terms of sex and age, provide evidence for the scale construct validity.

Moreover, it has been found that those adolescents who consumed alcohol frequently got higher scores in TAS, ES, DIS and SS Total Score but no difference in RNS was found. It seems that frequent alcohol users have a trend to seek novelty and risky experiences similar to the one observed in the occasional alcohol user group. Heavy episodic drinkers showed greater scores in ES, DIS, RNS and general SS. In this case, the only non-significant difference was for TAS (the only clear positive way of SS). DIS was the variable that better that best distinguished the HED group from non-HED group. This way of SS is a major concern for parents, school community and health public policies. Alcohol consumption is used in the West for disinhibition motives, especially in the social sphere (Arnett, 2012; Míguez, 2009). These results are important as the scale is supposed to discriminate different ways of using alcohol. That is why the differences observed here represent a contribution to the scale construct validity and show the possible utility of the scale.

We hypothesized that the differences found in our version may be due to cultural or environmental aspects. Other versions for adolescents or children (up to 15) from other cultures failed to find the same factors we have found. Thus, we assumed we have captured cultural aspects of sensation seeking. This construct is undoubtedly universal but the way it expresses itself depends on the ways and customs of a particular group. Identifying SS dimensions in particular groups is relevant for understanding this phenomenon in Latin American adolescents.

The adolescents studied showed a tendency to the search of sensations through forbidden and risky activities, disinhibited behavior, sports and novelty seeking. Thus, SS cannot be considered only a risk. As we developed in the introduction of this paper, SS represents an adaptive advantage. Going out to the world, to experience, to feel, to seek novelty, variety, sensations mean growing up, knowing, enriching, learning, challenging and daring. It is unimaginable an organism which rejects all kind of SS. Low levels of SS may be associated to anhedonia, apathy, abulia or, at least, it may be a very poor way of living, and even a risk as far as the organism cannot be provided with the necessary information or enrichment. So, from both ontogenetic and phylogenetic perspective, SS is a need and an advantage for most of primates.

Adolescents do not have many chances of practicing risk sports; these are very expensive activities and although they may be fond of them (as we can conclude from our results) they cannot reach them. Being TAS the only clear positive factor (at least socially accepted) of SS we asked ourselves which other protective factors are available for our adolescents when they have high levels of SS. The three other ways of SS (ES, RNS, DIS) are more likely in Latin American adolescents and the problem is that they seem to represent the negative side of SS. As we found in the present study, TAS gets the highest score; it means that they would like to practice risk sports but we do not know if they do so. In fact, we assumed a reduced proportion of adolescents are involved in this kind of activity. What happens then with those adolescents that having high levels of SS cannot accede to this socialized way of SS? In the Latin American population, drug consumption among adolescents is a growing health problem. It has reached levels never seen before: the age of initiation is decreasing, the prevalence is increasing and the way of consumption and the substances available are more dangerous; all this scenarios taking place in a socio-economic context characterized by extreme poverty. To these facts, which are similar all over Latin American, we must add the poor capacity of public policies to give any effective response to these problems and to integrate the great amount of excluded youths to the system (social, educational, work system). As a consequence, the main ways for adolescents to experience intense and new experiences and to canalize this need for sensations, is undoubtedly by disinhibited behaviour (parties, alcohol, sex) and by stimulation seeking (through drugs). Higher scores in TAS do not mean that they practise risky sports more frequently than they consume substances. The opposite is more likely. But it is showing an interesting data: considerable amount of adolescents would like to practise risky sports. Of course, drug abuse is a complex problem that has to be analyzed carefully and considering the influence of different kinds of determinants (socioeconomic, biological, behavioural, politic and even philosophical). But we cannot forget that at the psychobiological level, the best predictor is SS.

How to encourage positive ways of SS? These findings would have important implications for prevention programmes, since SS is related to a variety of behavioural and social problems not only in adolescence but also in adult population.

Gradually other scales are being developed as Arnett´s SSS (Arnett, 1994) or the brief scales devices by Stephenson, Hoyle, Palmgreen, and Slater (2003). But the creation of new scales does not mean that they are better (they show serious metric problems).

It is important to note some limitations of this study. We did not work with a random sample but convenience one. All subjects recruited were from Buenos Aires metropolitan area. But there is an enormous cultural variation in Latin America, even within each country. That’s why invariance of the present structure should be explored in several points of Latin America. The structure presented was the best solution of the many attempted because it combines the two conditions that were pursued: a coherent theoretical proposal and a solid psychometric structure. To achieve this, several items had to be removed. Which leads to another limitation of the present study: some content may be under-represented for some sensations seeking’s facets. Some items remain double loading which is a weakness of the scale, but removing any of these items led us to the loss of the 4 factor-structure. In future studies, the pool of items will be extended so as to be able to remove those with double loading without altering the theoretical model. Also in this line, in the future the dimensions proposed by Zuckerman’s model will be confirm by means of Confirmatory Factor Analysis (CFA) in an independent sample and the invariance of the structure will be explored when the scale is applied to a variety of samples: young athletes, chess players and musicians. On the other hand, the study of sensation seeking for adult population of Latin America is required, since the content of the current scale hardly reflect the sensation seeking in adult population.

Despite these limitations, this study showed that Zuckerman´s SSS is still valid and reliable even among Latin American Adolescents.
